# DotMapper: an open source tool for creating interactive disease point maps

**DOI:** 10.1186/s12879-016-1475-5

**Published:** 2016-04-12

**Authors:** Catherine M. Smith, Andrew C. Hayward

**Affiliations:** UCL Department of Infectious Disease Informatics, Farr Institute of Health Informatics Research, University College London, London, UK

**Keywords:** Tuberculosis, GIS, Web interface

## Abstract

**Background:**

Molecular strain typing of tuberculosis isolates has led to increased understanding of the epidemiological characteristics of the disease and improvements in its control, diagnosis and treatment. However, molecular cluster investigations, which aim to detect previously unidentified cases, remain challenging. Interactive dot mapping is a simple approach which could aid investigations by highlighting cases likely to share epidemiological links. Current tools generally require technical expertise or lack interactivity.

**Results:**

We designed a flexible application for producing disease dot maps using Shiny, a web application framework for the statistical software, R. The application displays locations of cases on an interactive map colour coded according to levels of categorical variables such as demographics and risk factors. Cases can be filtered by selecting combinations of these characteristics and by notification date. It can be used to rapidly identify geographic patterns amongst cases in molecular clusters of tuberculosis in space and time; generate hypotheses about disease transmission; identify outliers, and guide targeted control measures.

**Conclusions:**

DotMapper is a user-friendly application which enables rapid production of maps displaying locations of cases and their epidemiological characteristics without the need for specialist training in geographic information systems. Enhanced understanding of tuberculosis transmission using this application could facilitate improved detection of cases with epidemiological links and therefore lessen the public health impacts of the disease. It is a flexible system and also has broad international potential application to other investigations using geo-coded health information.

**Electronic supplementary material:**

The online version of this article (doi:10.1186/s12879-016-1475-5) contains supplementary material, which is available to authorized users.

## Background

Mapping is an important step in epidemiological disease investigations. Careful consideration of the spatial locations of cases on a map can prompt infectious disease outbreak investigations; highlight important relationships between cases including clusters defined by molecular typing techniques; generate hypotheses about transmission, and guide control measures [1]. Maps provide an easy-to-understand means of presenting data in context that is not as readily derived from tables of data or written reports, can be used as readily in high- and low-income settings, and can also be a powerful tool for advocacy.

Dot maps, for example, are a simple form of spatial data visualisation that can be used in preliminary exploration of data and may motivate more formal statistical cluster analysis. These maps display the locations of cases and can be colour coded to convey information on categorical variables such as demographics and risk factors. Production of dot maps for different time periods can describe the progression of the disease in space and time; and including data on contextual locations such as potential transmission venues can aid hypothesis generation.

In spite of these broad applications, this relatively simple means of visualising data is rarely used in real-time during disease outbreak investigations [1]. One of the barriers preventing regular production of dot maps is lack of flexibility in tools currently available, i.e., the ability to make rapid changes to the numbers of cases being displayed and the features highlighted. Specialised mapping software is also often expensive and may require trained personnel to operate it. Confidentiality considerations are also important when using patient location data, which can limit the application of this approach depending on local information governance procedures. A novel tool that facilitated the production of flexible, interactive dot maps with a user-friendly interface would therefore be useful within organisations with appropriate ethical approval to interrogate geographic information.

An example of a potential application of dot mapping involves investigation of cases of tuberculosis that are linked through molecular strain typing. In the United Kingdom, routine molecular strain typing by Mycobacterial Interspersed Repetitive Units – Variable Number Tandem Repeat (MIRU-VNTR) was introduced in 2010. This has led to increased understanding of the epidemiological characteristics of the disease and improvements in its control and diagnosis. For example, comparison of isolates taken from individuals at different time points has enabled estimation of the relative importance of reinfection and reactivation of disease [2]. Identification of cases infected with indistinguishable strain types has also been used to demonstrate or disprove active transmission between individuals, and to elucidate risk factors for transmission [3]. Monitoring strain types in circulation at a regional or national level, meanwhile, has enabled assessment of the effectiveness of control programmes and analysis of the global epidemiology of the disease [4]. Whole genome sequencing will further increase the degree of resolution with which strains can be distinguished as its use becomes more widespread [5].

However, the value of strain typing information for investigating molecular clusters of tuberculosis has been less clear. Molecular ‘clusters’ are groups of cases which share an indistinguishable molecular strain type and may therefore be part of the same chain of transmission. Cluster investigations aim to reduce public health impacts by detecting and diagnosing previously unidentified latently infected and active tuberculosis cases in these chains [6]. From January 2010 to December 2013, 81 % (16,602) of isolates for culture-confirmed cases in the United Kingdom were strain typed for at least 23 loci [7]. Over half (8,890) of these cases shared a strain type with at least one other case, and were therefore classified as part of a molecular cluster. A total of 1,854 distinct molecular clusters were identified, and initial guidelines required prospective investigation of all clusters that met certain thresholds [3]. An evaluation of the service in 2013 did not find that routine cluster investigation based on these criteria was effective or cost effective, and it was therefore discontinued [8]. Current recommendations state that local molecular cluster investigations should be conducted when deemed appropriate by public health professionals [3]. Novel tools would therefore be useful if they could assist with this process, both in the UK and internationally, by highlighting molecular clusters likely to share epidemiological links [1].

Tuberculosis cluster investigation through dot mapping could be implemented relatively easily where national case registers that include geographic data are maintained. The WHO recommends collection of address-level geographic information in electronic case registers for tuberculosis [9], and many countries including the United States and at least 23 in Europe also collect strain typing data on a routine basis [10, 11]. In the United Kingdom, for example, the Enhanced Tuberculosis Surveillance (ETS) system includes post code-level information for all cases, in addition to the routine molecular strain typing data. This means that cases can be plotted to a high degree of precision, and linked to other cases with the same molecular strain type.

### Current tools available

Geographic Information Systems (GIS) are databases designed for the input, management, analysis and display of geographically-referenced data [12]. One of the most commonly used GIS in epidemiology is ArcGIS/ArcView, a commercial package with many features ranging from production of simple dot maps to sophisticated analyses using spatial statistics [13]. QGIS is an open-source alternative to ArcGIS which shares some of the same features [14]. However, both of these programs require a degree of technical expertise to operate, even if the desired outputs are relatively simple.

Lightweight applications which are designed for a single function can provide an attractive alternative to broad GIS packages in some circumstances. For example, SaTScan is a program used for performing spatial scan statistics to identify significant clustering in data [15], and the European Centre for Disease Prevention and Control Map Maker (EMMa) is an online tool used to create maps of area-level data [16]. Both of these programs are free to use, have simple data requirements, and avoid the need to manually process geospatial data. A similar application for creation of dot maps would be useful to support disease cluster or outbreak investigations.

### Aims

We propose an interactive dot mapping application to aid epidemiological disease investigations by plotting locations of cases and their associated characteristics. The tool should be easy to operate without specialist GIS training, allow flexible data input and not require upload of patient identifiable information to the internet. It should also be written using open-source software and be freely available to download from a repository for further development or customisation.

Here, we describe our solution, DotMapper, and demonstrate its features and potential use with a case study based on molecular clusters of tuberculosis in London derived from ETS. Data presented are based on the characteristics of real clusters (for example the broad patterns in space and time), but anonymised by altering the exact characteristics including demographics, risk factors and spatial locations.

## Implementation

An interactive mapping tool was implemented using Shiny, a web application framework for the statistical software, R [17, 18]. Shiny applications are particularly useful for interrogation of sensitive data because they provide an interactive user interface but are run locally and therefore do not require upload of information to the internet. Interactive mapping was enabled through the R package Leaflet for the javascript library of the same name [19]. Base map tiles in the application are provided by OpenStreetMap, a free, editable world map, enabling visualisations produced to be shared without copyright restrictions [20]. Points are automatically colour coded according to levels of categorical variables using the package RColorBrewer to select colour palettes appropriate for cartography [21].

Additional features of the application include optional geocoding of postcodes or named geographic locations using the R package ggmap [22]; construction of epidemic curves using the packages ggplot2 and epitools [23, 24], and a summary data table comparing the characteristics of the selected cluster with the entire data set. Design of the application was inspired by the ‘SuperZip’ interactive visualisation by RStudio [25].

The application plots data of two types: cases (i.e., patient locations and associated characteristics) and, optionally, venues (i.e., other locations of interest such as clinics or potential sources of infection). The application was designed to be as flexible as possible to enable rapid plotting of data collected from different surveillance systems or surveys, although there are some requirements: Data, in the form of a .csv, .txt or .xls file, must be imported into R in “wide” format, with one row per individual; there must be a unique identifier for each individual case, venue, and case grouping; and categorical variables used for colour coding points should be the first columns of the data. The application was designed principally to display groups of cases at a local level, but it has been tested with data comprising up to 20,000 individual locations.

Scripts required for running DotMapper are provided as additional files with this article. They can also be downloaded from the GitHub repository (https://github.com/cathsmith57/DotMapper), which additionally includes a user guide, example data, and a link to a working demonstration of the application.

## Results and discussion

Here, we describe the main features of the application and illustrate their utility through a case study.

### Features of the application

The primary output of the application is an interactive map. We include three short screencast movies as additional files which demonstrate the interactive analyses of the application, and are discussed in more detail in the case study below. By default, the map displays the locations of the cases, colour coded according to the first categorical variable in the data set. Locations of contextual venues can be toggled on or off. The map can be panned and zoomed in and out to explore the data, and clicking on cases or venues produces a popup displaying further information. The application can be used to plot just one group of cases, for example in an outbreak situation, or to load multiple groups and compare their characteristics. Drop-down menus are used to select the group to display; to filter groups by size if necessary, and to change the variable being plotted.

Cases plotted can also be filtered by interactively selecting subsets of data: A date range slider is provided to select cases in any time period according to their notification date, and selecting “Subset” facilitates display of cases which satisfy selected combinations of characteristics of categorical variables. The “Reset groups” button returns the display to showing all cases.

Other tabs in the application display a summary data table and an epidemic curve. The data table presents the number and percentage of cases in the group according to each categorical variable. If multiple groups are included in the data it also displays totals and percentages for all cases, which can be used to assess whether patterns in the selected group reflect the overall epidemiology of the disease. Cases are plotted as a function of time using an epidemic curve. The time periods into which the cases are grouped in the epidemic curve can be switched between days, weeks, months, quarters and years, as appropriate for the specific disease being investigated.

### Case study

This case study presents data from three example molecular clusters of tuberculosis (denoted c1, c2 and c3) using altered and anonymised data of the same structure as that in ETS system.

Additional file 1 shows all cases in molecular cluster c1 displayed by ethnic group. There is a notable group of cases in the north east of the city which are of Pakistani ethnicity, whilst cases of other ethnicities appear to be more dispersed. Displaying only the cases in the first month of the cluster reveals that the initial cases were all in this spatially-constrained group of Pakistani ethnicity, and the cluster became more dispersed and affected different ethnic groups in later months. This visualisation can be used to generate hypotheses about transmission and potentially highlight a missed opportunity to for early control in a targeted population: The strain appears to have been transmitted amongst a distinct population group before being spread more widely in the community.

Assessment of molecular cluster c2 demonstrates the use of this tool in targeting interventions for specific risk populations (Additional file 2). Locations of cases in this cluster are displayed according to whether they have a history of homelessness, and locations of sheltered accommodation services are also shown. There appears to be an association with homelessness in the South central areas of London. The shelter in the south of the city may therefore be a suitable focus for interventions, such as screening by the mobile digital screening unit, Find and Treat [26].

Additional file 3 displays molecular cluster c3, comprised of individuals in a tight geographic cluster in the north central areas of the city, about half of whom were born in the United Kingdom and between the ages of 20 and 40. The epidemic curve shows that these cases all occurred within five quarters with numbers increasing recently, indicating a possible opportunity for control.

### Deployment

This application has been designed principally for use on local machines in a single-user context. We envisage public health officials and surveillance staff worldwide using the application to interrogate datasets without having to remove personal identifiable information from within secure systems. The application could also be used by researchers with appropriate data access rights. Installation of the software and its dependencies may be a challenge for some users who are less familiar with R, and we therefore propose disseminating via existing networks of local software “champions”.

The flexible nature of this tool permits a large scope for future developments. In its current form, a data set with any number of categorical variables can be plotted. However, bespoke versions of the tool with adaptations to specific data sets could be useful. For example, users may wish to customise categories into which continuous variables are divided, add additional information to popups, scale the size of the dots to represent multiple cases, or change the information displayed in the table. Such developments could be used locally or shared with professional networks through an online repository.

More advanced users could also extend the application by employing other features of the Leaflet library which are not implemented in the basic version of the tool, such as adding area data with polygons, overlaying images, enabling marker dragging, or using alternative base map tiles. An example of one such extension, overlaying a risk surface known as a geographic profile [27], is demonstrated at https://github.com/cathsmith57/geoprofileShiny.

## Discussion

This application enables rapid, interactive dot mapping of disease cases. It is intended to be used as a means for public health officials to visualise and interrogate geographically-referenced data without the need for specialist expertise in spatial epidemiology. In the context of investigation of molecular clusters of tuberculosis, we have demonstrated the ease with which the data presented on a map can be used to identify patterns in both space and time; be used to generate hypotheses about disease transmission pathways; identify cases of interest for future investigation, and guide potential control measures.

The application could also be used to improve understanding of tuberculosis more generally. Comparing the characteristics of cases in molecular clusters with non clustered cases, for example, may help to explain why some strains result in large outbreaks of disease. Cases managed by a particular clinic or those with multidrug-resistance, as opposed to sharing a specific molecular strain type, could also be plotted to assist with assessment of case loads. For example, the cohort review process used in the United States and United Kingdom involves regular appraisal of every case of tuberculosis [28, 29]. Assessment of maps of cases by experts with local knowledge could identify potential transmission venues, for example shelters for the homeless or venues associated with drug use. This could inform targeting of control measures such as contact tracing and screening.

Although we have focused on the example of tuberculosis here, the tool is easily adaptable to any geo-coded health data in any setting. Potential uses include investigation of outbreaks of gastrointestinal disease with a suspected foodborne origin, for which display of food outlets as contextual locations could be informative, and extracts from online surveys used to collect information on cases could be imported into the application with minimal data processing. The application could also be used to inform targeting of interventions in outbreaks of sexually transmitted infections. In these situations, the ability to filter cases with certain characteristics, for example according to sexual behaviours, could be particularly useful. Commissioners of services for non-communicable diseases may also find the application of use in identifying areas in greatest need of services by mapping locations of disease cases or events such as accidents. Finally, the application has potential uses in other areas of science, such as ecology, and in the commercial sector.

Another advantage of this application is its web interface, which provides intuitive features of interactive maps similar to those found across the internet with which users are likely to be familiar. This will therefore allow interrogation of spatial data in the context of epidemiological disease investigations to be separated from the need for expertise in the use of specialist GIS software. Use of the statistical software R, as opposed to a bespoke GIS package, will also help to streamline workflows: It allows mapping and epidemiological analyses to be conducted within the same software environment, eliminating the need to transfer data between software packages. This could be particularly advantageous in an outbreak situation, in which data is being updated on a regular basis. A future extension to this approach to mapping could be to create a mobile version of the application which could be used in the field to make live updates to data, which would currently only be possible with a laptop.

The main limitation of this application is that, as is common with open-source projects, it is not supported and therefore requires a degree of technical expertise amongst users to install and de-bug as necessary. This application is written in R and familiarity with this software will be needed for initial set up, however, the benefits of using the R framework include its large user-base and online community that will be able to provide assistance in many situations. Another potential limitation is the ease of sharing visualisations produced by this application within and between agencies, a number of which may be involved in an epidemiological disease investigation. Although screenshots, and screencast movies, as presented here, can be produced easily, this method clearly detracts from the interactive utility of the application.

It is also important to recognise the limitations of dot maps as an approach to epidemiological disease investigations. Whilst a useful first step for visualisation and hypothesis generation, dot maps do not account for the distribution of the underlying population and are therefore not a replacement for formal statistical tests of geographic clustering. Furthermore, these maps can be de-anonymising if displayed at a high zoom level. Avoiding the requirement to upload data to the internet gives this application the advantage of maintaining confidentiality, but incorporation of systems for sharing data would be beneficial for cross-agency exercises.

## Conclusion

In this study, we introduce DotMapper, an interactive mapping tool to support epidemiological investigations. The application is novel in providing rapid geographic displays of characteristics of cases in a user-friendly way without the need for specialised GIS software. It has broad applicability to epidemiological disease investigations any setting worldwide. In the context of tuberculosis control, we demonstrate its use in identification of common features of cases that are linked through molecular typing. Enhanced understanding of tuberculosis transmission using this application could lead to public health benefits by facilitating more appropriate targeting of services to diagnose and treat patients.

## Availability and requirements

Project name: DotMapper

Project home page: https://github.com/cathsmith57/DotMapper

Operating system(s): Platform independent

Programming language: R

Other requirements: R packages: shiny, leaflet, RColorBrewer, ggplot2, plyr, lubridate, zoo, epitools, tidyr, reshape2, ggmap

Licence: Apache License 2.0

Any restrictions to use by non-academics: N/A

### Availability of data and materials

Code is available to download from additional files with this manuscript and from the GitHub repository (https://github.com/cathsmith57/DotMapper).

## Additional files

Additional file 1: Screencast movie demonstrating use of the DotMapper interactive mapping application for investigation of example cluster c1. Base map tiles provided by OpenStreetMap © OpenStreetMap contributors. (MP4 12644 kb) Additional file 2: Screencast movie demonstrating use of the DotMapper interactive mapping application for investigation of example cluster c2. Base map tiles provided by OpenStreetMap © OpenStreetMap contributors. (MP4 20979 kb) Additional file 3: Screencast movie demonstrating use of the DotMapper interactive mapping application for investigation of example cluster c3. Base map tiles provided by OpenStreetMap © OpenStreetMap contributors. (MP4 12448 kb)

## Abbreviations

EMMa: European centre for disease prevention and control map maker
ETS: enhanced tuberculosis surveillance
GIS: geographic information systems
MIRU-VNTR: mycobacterial interspersed repetitive units – variable number tandem repeat
